# Microsatellite Instability in Endometrial Carcinoma by Immunohistochemistry, Association with Clinical and Histopathologic Parameters

**DOI:** 10.31557/APJCP.2019.20.9.2601

**Published:** 2019

**Authors:** Atif Ali Hashmi, Ghazala Mudassir, Rozina Nooreen Hashmi, Muhammad Irfan, Huda Asif, Erum Yousuf Khan, Syed Muhammad Abu Bakar, Naveen Faridi

**Affiliations:** 1 *Department of Histopathology, *; 4 *Department of Statistics, Liaquat National Hospital and Medical College, Karachi,*; 2 *Department of Pathology, Shifa College of Medicine, Islamabad,*; 3 *Department of Physiology, *; 5 *Medical student, CMH Institute of Medical Sciences, Multan, Pakistan. *

**Keywords:** Microsatellite Instability (MSI), endometrial carcinoma, Lynch syndrome, hereditary

## Abstract

**Objective:**

We aimed to investigate the frequency of microsatellite instability (MSI) in endometrial carcinoma in our population and its association with clinico-pathologic features.

**Methods:**

A total of 126 cases of primary endometrial carcinoma were included in the study that underwent surgical resections. All slides of these cases were reviewed and representative paraffin fixed tissue blocks were selected for MLH1, MSH2, MSH6 and PMS2 IHC staining. IHC expression was categorized into five groups: no loss of expression; loss of expression of all four antibodies; combined loss of MLH1/PMS2; combined loss of MSH2/MSH6; and isolated loss of MLH1. Pathological records of all cases were retrieved from patient files.

**Result:**

Abnormal expression of MSI was noted in 56 cases (44.4%) among which 16 cases showed loss of nuclear expression of all markers, 34 cases showed loss of MLH1/PMS2 expression, 4 cases showed loss of MSH2/MSH6 while only 2 cases revealed isolated loss of MLH. Personal and family history suggestive of inherited cancer susceptibility was revealed in 11 cases most of which were associated with MSH2/MSH6 loss. Significant association of MSI expression was found with tumor stage and personal/family history of endometrial/colon cancer.

**Conclusion:**

A high frequency of endometrioid cancers in our study showed abnormal expression of MSI markers, most of which depicted MLH1/PMS2 loss and were not associated with inherited cancer susceptibility. On the other hand, a minority of cases showed loss of all MSI markers or MSH2/MSH6 loss and were significantly associated with family/personal history of cancer. Therefore, we suggest that epigenetic changes in MLH1 locus may be a predominant pathway of tumorigenesis in our population rather than inherited mutation of MSI genes; however more large scale studies with genetic testing are required to validate this observation.

## Introduction

Microsatellite Instability (MSI) has emerged as one of the major pathways in endometrial carcinogenesis (McMeekin et al., 2016). Microsatellites are short segments of repetitive DNA sequences found predominantly in non-coding DNA of human genome. MSI leads to an increased propensity to develop changes in the number of repeat elements as compared with normal tissue due to DNA repair errors made during replication (Kolodner and Marsischky, 1999; Esteller et al., 1998). High levels of MSI called as MSI-high measured in the DNA of tumor cells compared with normal tissue DNA occur due to one of two possible causes. These include germline or sporadic mutation in at least one of the DNA mismatch repair enzymes (MLH1, PMS2, MSH2, and MSH6) and epigenetic silencing due to *MLH1* gene promoter hypermethylation. 

**Table 1:** Expression of Microsatellite Instability (MSI) Markers in Endometrial Carcinoma and Its Association with Clinicopathologic and Prognostic Parameters

	n (%)						
	Intact expression of all markers (n=70)	Loss of expression of all markers (n=16)	MLH1/PMS2 Loss of expression (n=34)	MSH2/MSH6 Loss of expression (n=4)	Isolated MLH1 loss of expression (n=2)	Total (n=126)	P-value
Age Group							
<50 yrs	24 (34.3)	7 (41.2)	10 (30.3)	2 (50)	0 (0)	43 (34.1)	0.784**
>50 yrs	46 (65.7)	10 (58.8)	23 (69.7)	2 (50)	2 (100)	83 (65.9)	
Menopausal Status							
Pre Menopausal	10 (14.3)	3 (17.6)	4 (12.1)	0 (0)	0 (0)	17 (13.5)	0.946**
Post Menopausal	60 (85.7)	14 (82.4)	29 (87.9)	4 (100)	2 (100)	109 (86.5)	
Tumor (T) Stage							
T1	44 (62.9)	8 (47.1)	25 (75.8)	2 (50)	2 (100)	81 (64.3)	0.012*
T2	13 (18.6)	7 (41.2)	6 (18.2)	0 (0)	0 (0)	26 (20.6)	
T3	13 (18.6)	0 (0)	2 (6.1)	2 (50)	0 (0)	17 (13.5)	
T4	0 (0)	2 (11.8)	0 (0)	0 (0)	0 (0)	2 (1.6)	
Nodal (N) Stage							
N0	70 (100)	15 (88.2)	33 (100)	2 (50)	2 (100)	122 (96.8)	0.000*
N1	0 (0)	2 (11.8)	0 (0)	0 (0)	0 (0)	2 (1.6)	
N2	0 (0)	0 (0)	0 (0)	2 (50)	0 (0)	2 (1.6)	
FIGO Stage							
Stage I	41 (58.6)	8 (47.1)	25 (75.8)	2 (50)	2 (100)	78 (61.9)	0.014*
Stage II	15 (22.9)	7 (41.2)	6 (18.2)	0 (0)	0 (0)	29 (23)	
Stage III	13 (18.6)	0 (0)	2 (6.1)	2 (50)	0 (0)	17 (13.5)	
Stage IV	0 (0)	2 (11.8)	0 (0)	0 (0)	0 (0)	2 (1.6)	
FIGO Grade							
Grade I	30 (42.9)	7 (41.2)	15 (45.5)	2 (50)	2 (100)	56 (44.4)	0.824**
Grade II	33 (47.1)	10 (58.8)	16 (48.5)	2 (50)	0 (0)	61 (48.4)	
Grade III	7 (10)	0 (0)	2 (6.1)	0 (0)	0 (0)	9 (7.1)	
Cervical Invasion							
Present	23 (32.9)	9 (52.9)	6 (18.2)	2 (50)	0 (0)	40 (31.7)	0.077**
Absent	47 (67.1)	8 (47.1)	27 (81.8)	2 (50)	2 (100)	86 (68.3)	
Adnexal Involvement							
Present	10 (14.3)	0 (0)	0 (0)	2 (50)	0 (0)	12 (9.5)	0.007*
Absent	60 (85.7)	17 (100)	33 (100)	2 (50)	2 (100)	114 (90.5)	
Lymphovascular Invasion							
Present	4 (5.7)	0 (0)	2 (6.1)	0 (0)	0 (0)	6 (4.8)	0.796**
Absent	66 (94.3)	17 (100)	31 (93.9)	4 (100)	2 (100)	120 (95.2)	
Medical/family history of endometrial/colon cancer							
Present	0 (0)	7 (43.8)	2 (5.9)	2 (50)	0 (0)	11 (8.7)	0.000*
Absent	70 (100)	9 (56.3)	32 (94.1)	2 (50)	2 (100)	115 (91.3)	
Recurrence							
Yes	12 (17.1)	6 (37.5)	4 (11.8)	1 (25)	0 (0)	23 (18.3)	0.218**
No	58 (82.9)	10 (62.5)	30 (88.2)	3 (75)	2 (100)	103 (81.7)	

*P-value is significant at 95% confidence interval

**Figure 1:**
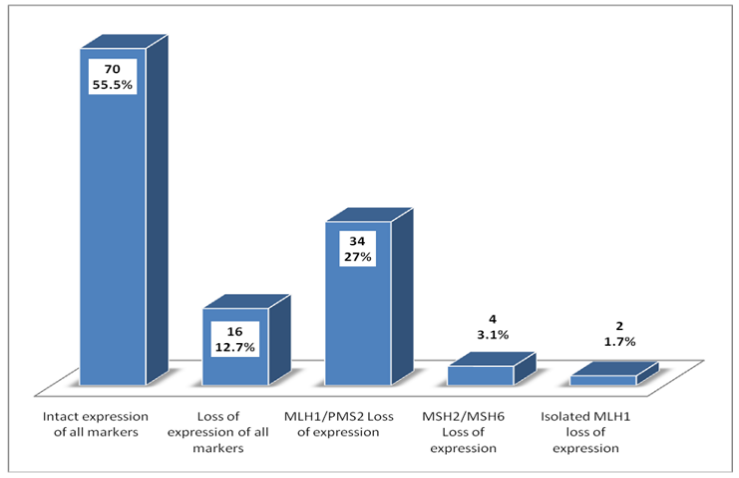
Frequency of Expression of Microsatellite Instability (MSI) Markers in the Studied Population

**Figure 2:**
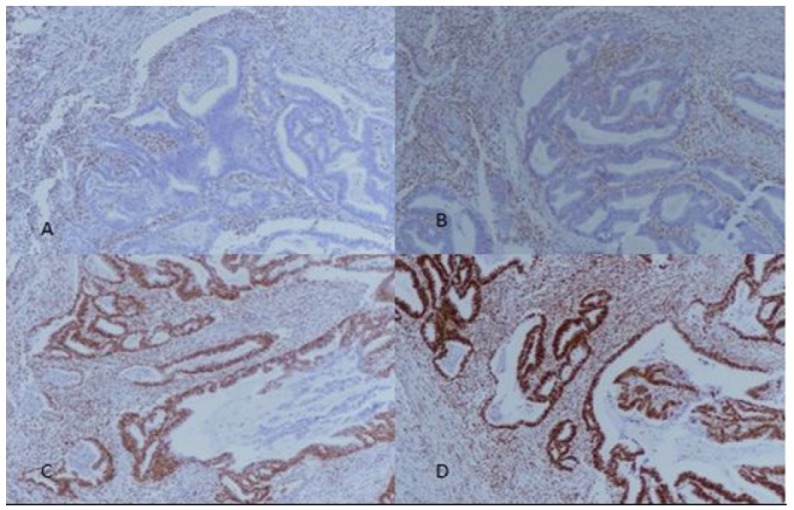
A+B, Loss of *MLH1* and *PMS2* Expression Respectively (100X Magnification); C+D, Intact Expression of *MSH2* and *MSH6* (100X Magnification). Stromal cells and normal endometrial glands serve as internal controls

**Figure 3:**
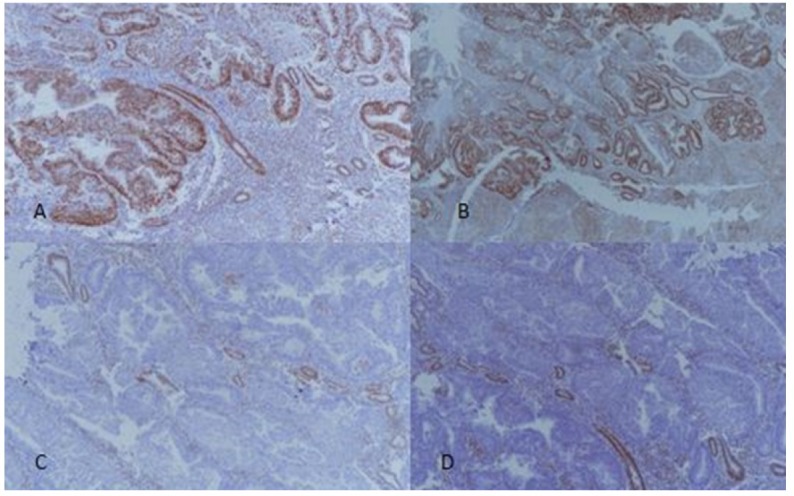
A, MSH2 – Intact Expression, 100X Magnification; B, MSH6 – Intact Expression, 100X Magnification; C, PMS2 – Loss of Expression, 100X Magnification; D, MLH1 - Loss of Expression, 100X Magnification

MSI is usually found in endometrioid endometrial cancers. On the other hand, non-endometrioid endometrial cancers show genetic instability at chromosomal level due to primary defects in p53 gene, rather than microsatellite level. MSI in endometrial cancers secondary to sporadic mutations or epigenetic silencing has been estimated to occur in approximately 15-20% of endometrial cancers; whereas, germline mutations account for approximately 5% of endometrial cancers (Gruber and Thompson, 1996; Dunlop et al., 1997). These germline mutations in mismatch repair genes lead to a site specific genetic predisposition of cancers referred to as lynch syndrome or hereditary non-polyposis colorectal cancer (HNPCC) syndrome. Apart from an increased risk of colorectal carcinoma, women with HNPCC have a substantial increase in risk of developing endometrial cancer (40-60% life time risk with MLH1 and MSH2 mutations, 71% risk with MSH2 mutations) (Aarnio et al.,1999; Hendriks et al., 2004; Watson et al., 2008). Risk for other cancers is also increased including lower urinary tract, ovary, stomach and small bowel (Watson et al., 2008). Although genetic testing remains gold standard for detection of MSI, loss of expression of mismatch repair enzymes MLH1, PMS2, MSH2 and MSH6 by immunohistochemistry (IHC) may be used as a surrogate marker for MSI (Hashmi et al., 2018). To date, there is limited data to suggest that MSI-high endometrial cancers are prognostically favorable or worse as compared to chromosomally instable endometrial cancers. Furthermore, histologic parameters of these tumors has not yet been studied in our population, therefore we aimed to investigate the frequency of MSI in endometrioid endometrial carcinoma in our population and its association with clinico-pathologic features using a four antibody panel.

## Materials and Methods

A total of 126 cases of primary endometrial carcinomas were included in the study. All patients underwent surgical resections between January 2014 and December 2015 at Liaquat National Hospital, Karachi, Pakistan. All cases were biopsy proven. Patients with history of pre-operative chemotherapy or radiation therapy were excluded from the study. Moreover, cases with non-endometrioid histology were also excluded. Medical reports of the patients were reviewed and patients were called to reveal personal and family history of cancers suggestive of inherited cancer susceptibility. The study was approved by the hospital ethical committee.

All slides of these cases were retrieved and reviewed. Then representative paraffin fixed tissue blocks were selected for IHC staining that showed both tumor and adjacent non-cancerous endometrium. Immunohistochemistry with MLH1, MSH2, MSH6 and PMS2 was performed by using Dako envision method on representative paraffin fixed tissue blocks according to manufacturers protocols. IHC was performed manually in batches of 10 with positive and negative controls run along each batch. Normal colonic tissue is used as positive control. Known case of microsatellite instable colorectal cancer is used as negative control. Microarrays were not used. Any nuclear staining in tumor cells was taken as no loss of expression; on the other hand, loss of expression was interpreted when there was no nuclear staining for any one of the antibody as per College of American Pathologists (CAP) guidelines. IHC expression was then categorized into five groups: no loss of expression; loss of expression of all four antibodies; combined loss of MLH1/PMS2; combined loss of MSH2/MSH6; and isolated loss of MLH1as shown in Figures 1 and 2. Pathological records of all cases were retrieved from patient files. In addition, all retrieved slides were independently reviewed by two histopathologists.

*Statistical analysis*

Statistical package for social sciences (SPSS 21) was used for analysis. Mean and standard deviation were calculated for quantitative variables. Frequency and percentage were evaluated for qualitative variables. Chi square test was applied to determine association. P-value of ≤0.05 was taken as significant.

## Results

Mean age of the patients included in the study was 55.10 (34-70) years. Majority of the patients were above 50 years of age (65.9%) and most of the patients were post-menopausal (86.5%). FIGO stage I (T1N0) was the most frequent stage at presentation (61.9%) and cervical or adnexal involvement was noted in a minority of cases (31.7% and 9.5% respectively). Similarly, a minority of cases were found to be at high grade/ grade III (7.1%). Overall 11 cases had personal or family history of cancers, out of which 4 patients had family history of endometrial carcinoma, 3 had family history of colorectal carcinoma, 2 had family history of pancreato-biliary cancer and 2 had synchronous colorectal carcinoma. Abnormal expression of MSI was noted in 56 cases (44.4%) among which 16 cases show loss of expression in all markers, 34 cases showed *MLH1/PMS2* loss of expression, 4 cases showed *MSH2/MSH6* loss of expression and only 2 cases showed isolated *MLH1* loss of expression as shown in figure 3. Most of the cases with personal or family history of cancers showed *MSH2/MSH6 *loss as shown in Table 1 (P value <0.001). Similarly, significant association of MSI expression was found with tumor stage, nodal Stage, FIGO stage, adnexal involvement and medical/family history of endometrial/colon cancer as shown in Table 1. Cases with loss of expression of all *MSI* markers were found to be at higher TNM/ FIGO stage compared to cases showing intact expression of all markers; however no significant association of MSI was seen with tumor grade or tumor recurrence. 

## Discussion

In the current study, we evaluated MSI status of endometrial cancers in Pakistani patients. Endometrial cancers are a major cause of morbidity and mortality in SouthAsia (Tanvir et al., 2014; Hashmi et al., 2018). To our knowledge this is the first study evaluating MSI status in our population. Abnormal expression of MSI in our population was turned out to be 44% which is quite high compared to studies conducted in other parts of the world. Frequency of MSI+ endometrial cancers is quite variable. A study conducted in USA involving 473 cases of endometrial cancers revealed 20% MSI+ tumors. They found MSI+ tumors to be associated with more favorable outcome despite being linked to prognostically poor histologic parameters like pathologic stage and grade (Black et al., 2006). It has been well established that, MSI is specifically linked to endometrioid histology (Catasus et al., 1998), therefore we excluded non-endometrioid endometrial cancers from our study. An NRG oncology/gynecology group study, in a large series of cases, assigned 1,024 cases into four MMR classes and found that MMR defects were linked to poor prognostic factors like higher grade, myometrial invasion and lymphovascular invasion. On the other hand, there was no significant difference in survival of these patients and they suggested that immune surveillance associated with MMR defects may counteract the effects of negative prognostic factors in these patients (Mc Meekin et al., 2016). Similarly in another study MSI- high status was detected in 15.6% endometrial cancers while no statistically significant differences between patients with MSI-high and MSI stable tumors was found after stage, histology and tumor grade adjustment on multivariate analysis (Kanopiene et al., 2014). Our results are concordant with these findings as we found association of MSI+ endometrial cancers with high TNM/ FIGO stage, however there was no association found with disease free survival. Histologic features of MSI high tumors include poor differentiation, intra-tumoral and peri-tumoral lymphocytic reaction in colorectal cancers; however they are not validated in endometrial cancers.

In our study, tumors with dMMR status accounted for 44% of total cases. Only a few studies have been conducted in SouthAsia evaluating MSI in endometrial cancers. A study conducted in India revealed 30% frequency of MSI in endometrial cancers; however they didn’t excluded non-endometrioid cancers from their study, which may be the reason of this difference (Kunitomi et al., 2017). Concurrent loss of MLH1/PMS2 was the most common pattern of abnormal protein expression followed by loss of all markers in our study. A germ-line mutation in one of the* MMR* genes is the cause of dMMR in patients with HNPCC (Lynch syndrome) (Zhang et al., 2006). These tumors show high levels of MSI (MSI-H). However a high proportion of sporadic endometrial cancers with no family history also exhibit MSI; on the other hand in sporadic cases, mutation of MMR genes are infrequent whereas biallelic hypermethylation of promotor MLH1 appears to be most important mechanism for inactivation of MMR genes. In a study it was established that 77% of MSI positive cancers showed MLH1 promotor hypermethylation. MLH 1 promotor methylation was associated with loss of* MLH1* expression with or without concurrent loss of *PMS2* expression. On the other hand, loss of *MSH2/MSH6* expression was found to be infrequent in these cases. Furthermore, they suggested that MSH2 loss may be linked preferentially to inherited forms of endometrial cancer (cases which are associated with HNPCC) (Simpkins et al.,1999). These findings correlate well with our study in which MLH1/PMS2 loss was the most common pattern while loss of expression of MSH2/MSH6 was seen in a minority of cases and MSH2 loss was significantly associated with inherited cancer susceptibility. 

Another significance of MSI testing is therapeutic benefit of anti-PDL therapy in MSI associated endometrial cancers. Role of immune therapy is increasing in many human cancers which express *PDL1*. It has been proposed that MSI associated endometrial cancers have better response to anti-PDL therapy compared to microsatellite stable endometrial cancers (Howitt et al., 2015).

Our study can be viewed with a few limitations like lack of availability of molecular studies, however the findings of our study may be clinically relevant and may open a door to more large scale studies evaluating genetic makeup of endometrial cancers in our population to validate these findings. High frequency of endometrioid cancers in our study showed abnormal expression of MSI markers, most of which depicted MLH1/PMS2 loss and were not associated with inherited cancer susceptibility. On the other hand, a minority of cases showed loss of all MSI markers or MSH2/MSH6 loss and were significantly associated with family/personal history of endometrial/colon cancer. Therefore, it can be suggested that epigenetic changes in MLH1 locus may be a predominant pathway of tumorigenesis in our population rather than inherited mutation of MSI positive endometrial cancers, however more large scale studies with genetic testing is required to validate this observation. 

## Data Availability

Please contact author for data requests.
